# Supplementary material for: Identification of PCPE-2 as the endogenous specific inhibitor of human BMP-1/tolloid-like proteinases
Source: Nat Commun. 2023 Dec 4;14:8020. doi: 10.1038/s41467-023-43401-0 (PMC10696041; doi:10.1038/s41467-023-43401-0)
Supplement: Supplementary file 1 — Supplementary Information [file 41467_2023_43401_MOESM1_ESM.pdf]

Supplementary Information for

Identification of PCPE-2 as the endogenous specific inhibitor of human BMP-1/tolloid-like proteinases

Sandrine Vadon-Le Goff, Agnès Tessier, Manon Napoli, Cindy Dieryckx, Julien Bauer, Mélissa Dussoyer, Priscillia Lagoutte, Célian Peyronnel, Lucie Essayan, Svenja Kleiser, Nicole Tueni, Emmanuel Bettler, Natacha Mariano, Elisabeth Errazuriz-Cerda, Carole Fruchart Gaillard, Florence Ruggiero, Christoph Becker-Pauly, Jean-Marc Allain, Leena Bruckner-Tuderman, Alexander Nyström, Catherine Moali*

***Corresponding author. Email:** catherine.moali@ibcp.fr

This PDF file includes:

Supplementary Methods
Supplementary Table 1
Supplementary Figures 1-16
Supplementary References

Supplementary Methods

Source of proteins

Human BMP-1 (fused to a C-terminal Flag tag[1,2]), human PCPE-1 (fused to a C-terminal 8His tag[2] or native form[3]), its NTR domain (obtained by limited trypsinolysis[4]), mini-procollagen III (fused to a N-terminal c-myc tag[3]), the C-propeptide of procollagen III (starting at aspartate 1222; no tag[5]) and pNα1(V)[6] were produced in human embryonic kidney (HEK) 293-EBNA cells and purified as previously described. CPIII-Long[7] and mini-procollagens I and II[8] were obtained and purified from 293-T cells. CUB1CUB2 from PCPE-1 (fused to a C-terminal 6His tag) was produced in 293-F cells[9]. Rat betaglycan was a kind gift of Fernando Lopez-Casillas (Universidad Nacional Autonoma de Mexico, Instituto de Fisiologia Celular, Mexico) and was prepared with the baculovirus/High five cells system[10]. Recombinant human meprin α and meprin β were also expressed in this system and were purified and activated as described previously[11,12]. TSP-1 was purified from human platelets as described in Anastasi *et al.*[13]. Finally, human chordin (ref. 758-CN/CF), human endorepellin (ref. 2364-ER), the ectodomain of human LDLR (ref. 2148-HP) and the ectodomain of human CD99 fused to the Fc domain of IgG1 (ref. 3968-CD) were purchased from BioTechne.

The catalytic domain of BMP-1 (BMP-1[cat]) was produced in *E. coli*, following the protocol reported in Mac Sweeney *et al.*[14] with some modifications. In brief, the optimized sequence encoding the human BMP-1 catalytic domain (A121-A321) was ordered from GeneArt without purification tag and subcloned between the *NdeI* and *BamHI* restriction sites of pET24a (Novagen). The BMP-1cat plasmid was transformed in BL21(DE3) Star competent cells and expressed in ZYP-5052 auto-inducing medium, using a two-step standardized procedure (4 h at 37 °C to reach glucose depletion and 16 h at 20 °C). The cell pellet was then resuspended in 50 mM Tris HCl pH 8.0, 5 mM EDTA and 5 mM benzamidine, cells were lysed and the insoluble fraction was washed as described[14]. Inclusion bodies solubilized in 50 mM Tris HCl pH 8.5, 6 M guanidinium chloride and 100 mM DTT were centrifuged and the supernatant was dialyzed against the same buffer without DTT. Solubilized proteins were then diluted to 100 µg/ml in refolding buffer (50 mM Tris HCl pH 8.5, 125 mM NaCl, 1 mM $CaCl_2$, 10 µM $ZnCl_2$, 0.8 M L-arginine, 0.1 mM cysteine and 1 mM cystine) and refolded by gentle stirring at 4 °C for 7 days. After dialysis in buffer A (25 mM Tris HCl pH 7.5, 125 mM NaCl, 1 mM $CaCl_2$, 10 µM $ZnCl_2$), the refolded protein solution was supplemented with 1.8 M ammonium sulfate and purified on Phenyl Sepharose 6 Fast Flow (Cytiva) equilibrated in buffer A with 1.8 M

ammonium sulfate. After elution in buffer A, the protein was dialyzed again against the same buffer to completely eliminate ammonium sulfate. Finally, the protein was concentrated with Vivaspin 6 PES (5 kDa MWCO, Sartorius) and stored at -80 °C. The production yield was around 4.5 mg of BMP-1[cat] per liter of culture.

mTLD cDNA (gift from Prof. A. Sieron, Medical University of Silesia, Katowice, Poland) was inserted into the mammalian expression vector pCEP4 with a C-terminal V5-8His tag. HEK 293-EBNA cells were stably transfected using nanofectin (PAA) as the transfection agent and hygromycin B (Sigma) as selection agent. mTLD was purified by affinity chromatography (Ni-NTA) followed by size exclusion chromatography, as previously described[15].

Macros used for the analysis of TEM pictures with ImageJ

Heart

```
////////////////////////////////////////////////////////////////////////

//Macro used to measure fibril diameter and nearest-neighbor distance (NND) from
//HIGHLY CONTRASTED EM images with a scale > 2 pixels / nm

//Starts with an open image with a ROI overlayed where detection must be performed
//@Jacques Brocard for LBTI 2022

////////////////////////////////////////////////////////////////////////


//---INITIALIZATION
run("Select All");
percent_area=40; //percentage of fibers area within ROI
min_radius=12.5; //min radius of fibers = 12.5 nm
t=getTitle();
dir=getDirectory("image");
getPixelSize(unit, pixelWidth, pixelHeight);
run("Duplicate...", "title=temp");
run( "Properties...", "unit=pixel pixel_width=1 pixel_height=1 voxel_depth=1");
min_radius=min_radius/pixelWidth; //min radius of fibers in pixels

//--- THRESHOLDING
run("To ROI Manager");
setAutoThreshold("Percentile");
roiManager("Select", 0);
setThreshold(0, percenthreshold(percent_area));
setOption("BlackBackground", true);
run("Convert to Mask");

//--- SMOOTHING
run("Options...", "iterations=2 count=1 black pad do=Erode");
run("Fill Holes");
run("Options...", "iterations=2 count=1 black pad do=Erode");
run("Analyze Particles...", "size=100-Infinity show=Masks in_situ");
run("Options...", "iterations=4 count=1 black pad do=Dilate");
run("Fill Holes");
run("Options...", "iterations=2 count=1 black pad do=Close");

//--- INDIVIDUAL FIBER SEGMENTATION based on ultimate points
run("Duplicate...", "title=voronoi");
run("Ultimate Points");
setThreshold(min_radius,255);
setOption("BlackBackground", true);
run("Convert to Mask");
roiManager("Select",0);
```

```
run("Analyze Particles...", "size=1-Infinity show=Masks in_situ");
run("Options...", "iterations=30 count=1 black pad do=Dilate");
run("Voronoi");
setThreshold(1,255);
setOption("BlackBackground", true);
run("Convert to Mask");
imageCalculator("Subtract", "temp","voronoi");
close("voronoi");
roiManager("Select",0);
run("Analyze Particles...", "size="+(PI*min_radius*min_radius)+"-Infinity add clear");
//--- SAVE CORRESPONDING ROIS
roiManager("Save", dir+substring(t,0,lengthOf(t)-4)+".zip");

//--- NND MEASUREMENTS
run("Set Measurements...", "area mean centroid redirect=None decimal=3");
roiManager("Measure");
Area=Table.getColumn("Area");
Radius=newArray(Area.length+1);
X=Table.getColumn("X");
Y= Table.getColumn("Y");
NND=newArray(X.length+1);
close("temp");
close("Results");
for (i=0;i<Area.length;i++){
	Radius[i]=sqrt(Area[i]/PI);
	mini=9999;
	for (j=0;j<Area.length;j++){
		dist=sqrt((X[j]-X[i])*(X[j]-X[i])+(Y[j]-Y[i])*(Y[j]-Y[i]));
		if ((dist>0) && (dist<mini)) mini=dist;
	}
	NND[i]=mini;
}

//--- LOG WRITING
print("Num \t Radius ("+unit+") \t NND ("+unit+")");
for (i=0;i<Area.length;i++){
	print((i+1)+"\t"+(Radius[i]*pixelWidth)+"\t"+(NND[i]*pixelWidth));
}
saveAs("Text",dir+substring(t,0,lengthOf(t)-4)+".txt");
close("Log");
roiManager("Show All");

function percenthreshold(percentage){
	nBins = 256;
	resetMinAndMax();
	getHistogram(values, counts, nBins);
	nPixels = 0;
	for (i = 0; i<counts.length; i++)
	  nPixels += counts[i];
	nBelowThreshold = nPixels * percentage / 100;
```

```
    i=0; sum=0;
    while (sum < nBelowThreshold){
        sum=sum+counts[i];
        i++;
    }
    return values[i];
}
```

Tendon

```
////////////////////////////////////////////////////////////////////////
/*
Date : 10/02/2015
Author : Denis Ressnikoff
version : 1.0

Macro used to measure the geodesic diameter of collagen fibrils on TEM pictures of tendons
with a fixed calibration (1 px = 1.0041 nm)

Necessary Plugin(s):
	*ij_Geodesics (https://www.pfl-cepia.inra.fr/index.php?page=ijGeodesics_en)

*/
//////////////////////////////////////////////////////////////////////

//initialisation

run("Set Measurements...", "area limit display redirect=None decimal=2");
run("Colors...", "foreground=black background=white selection=green");

if (isOpen("ROI Manager")) {
	selectWindow("ROI Manager");
	run("Close");
}

if (isOpen("Log")) {
	selectWindow("Log");
	run("Close");
}

if (isOpen("Results")) {
	selectWindow("Results");
	run("Close");
}

rep = getDirectory("image");
img = getTitle();

//calibration
run("Set Scale...", "distance=1.0041 known=1 pixel=1 unit=nm global");
run("Duplicate...", "title=count");

//post-treatment filtering
run("Gaussian Blur...", "sigma=5");
//segmentation
setAutoThreshold("Huang");
setOption("BlackBackground", false);
run("Convert to Mask");
```

```
run("Watershed");

//run("Options...", "iterations=5 count=4 edm=Overwrite do=Open");
run("Fill Holes");

//analyse particules
run("Analyze Particles...", "size=750-Infinity circularity=0.4-1.00 show=[Count Masks]
exclude clear add");

//diameter
run("Geodesic Diameter", "label=[Count Masks of count] distances=[Borgefors (3,4)] show
image=["+img+"]");

//saving
saveAs("Results", rep+img+".xls");
close("Count Masks of count");
close("count");

run("Flatten");
rename(img+" mesure");
saveAs("Jpeg", rep+img+" mesure.jpg");
close(img);
//close(img+" mesure.jpg");
run("Tile")
```

Supplementary Table 1: Primers used in qRT-PCR experiments.

Primer	Sequence
PCOLCE-for	AACTACACCAGACCCGTGTTC
PCOLCE-rev	CGGAATGAGAGGGACACAGT
Pcolce-for	CTTCGGGCACTGAGCACCA
Pcolce-rev	ACCTGGTTTGAGGGTGCAAT
PCOLCE2-for	ATAGTCCACCTGCGCCAAT
PCOLCE2-rev	ACCCGTGGTTACAGGGAATG
Pcolce2-for	CATGTGGCGGCATTCTTACC
Pcolce2-rev	TGCCCTCAGGAACTGTGATT
Bmp1-1-for	TGCGTGTGGAGTTCAAGTCT
Bmp1-1-rev	CCGAGGTGAGCTTCCTGAGT
Bmp1-3-for	GTACGTGGGCTATCTCCAGC
Bmp1-3-rev	GCTCAGGCTTCTTACTGCCA
Tll1-for	GGCTGGAGTTCTTACATCTACG
Tll1-rev	CTTATCTCCCCTCCACAAATCG
Bmp2-for	CGGACTGCGGTCTCCTAA
Bmp2-rev	TGCTGATGATCGAGACCGTG
Tgfb1-for	TCACTGGAGTTGTACGGCAG
Tgfb1-rev	GTTTGGGGCTGATCCCGT
GAPDH-for	GGCCTCCAAGGAGTAAGACC
GAPDH-rev	AGGGTCTACATGGCAACTG
Gapdh-for	TTGATGGCAACAATCTCCAC
Gapdh-rev	CGTCCCGTAGACAAAATGGT
Rpl13a-for	ATCCCTCCACCCTATGACAA
Rpl13a-rev	GCCCCAGGTAAGCAAACTT

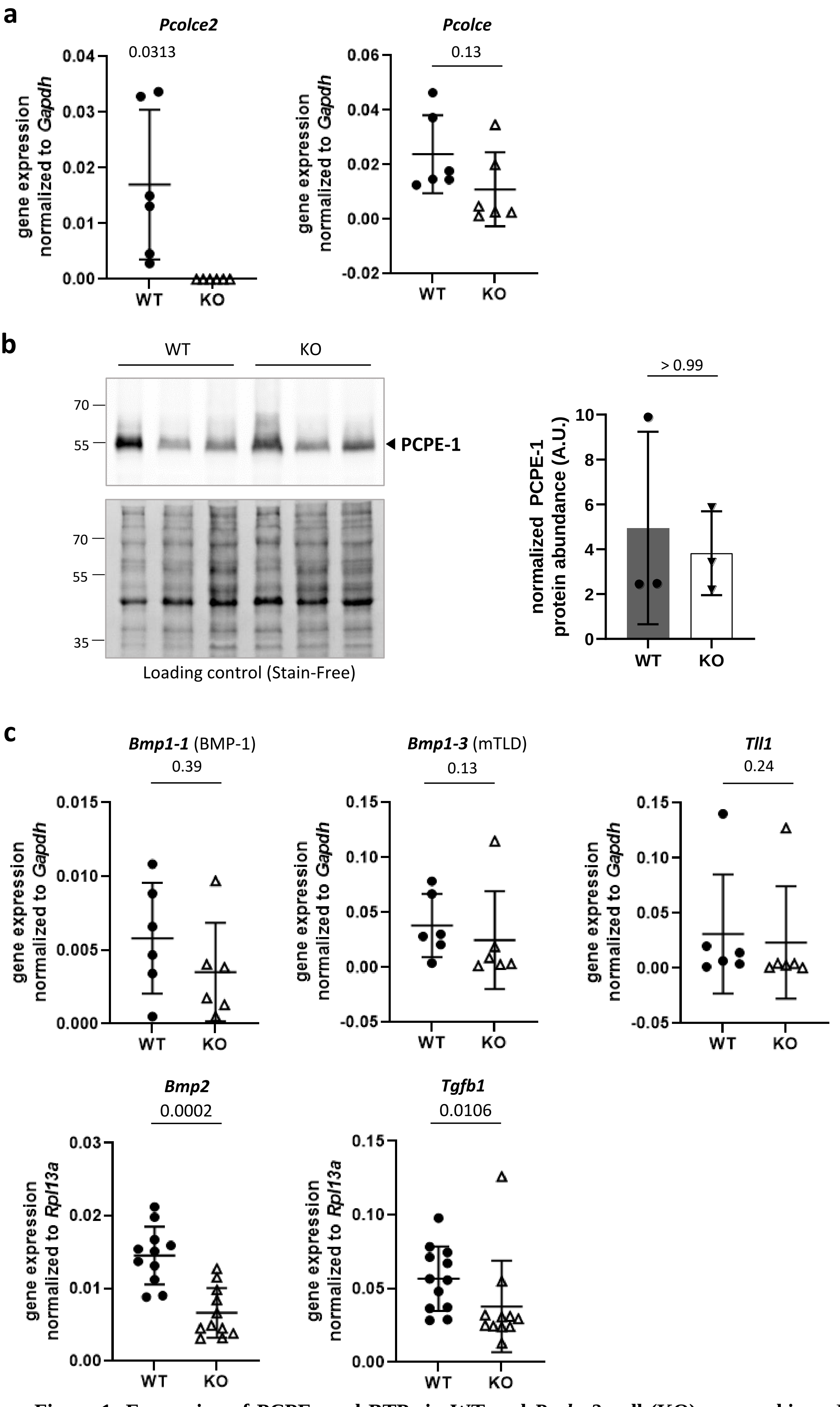

Supplementary Figure 1: Expression of PCPEs and BTPs in WT and *Pcolce2*-null (KO) mouse skin. a Relative mRNA quantification of *Pcolce* and *Pcolce2* by qRT-PCR. Means ± SD (n = 6 mice/genotype; Mann-Whitney test for *Pcolce*; Wilcoxon signed rank test for *Pcolce2*). **b** Detection of PCPE-1 by Western blotting (with AF2239 antibody) in skin lysates of WT and *Pcolce2* KO mice (12 % acrylamide gel; reducing conditions). Normalization was by total protein amount quantified after Stain-Free detection (means ± SD; Mann-Whitney test; n = 3 mice/genotype). **c** Relative mRNA quantification of *Bmp1-1 and Bmp1-3* (two splice variants of the *Bmp1* gene; n = 6 mice/genotype), *Tll1* (n = 6 mice/genotype), *Bmp2* (n = 11 mice/genotype) and *Tgfb1* (n = 12 for WT mice and 11 for KO) mRNA by qRT-PCR (Mann-Whitney test).

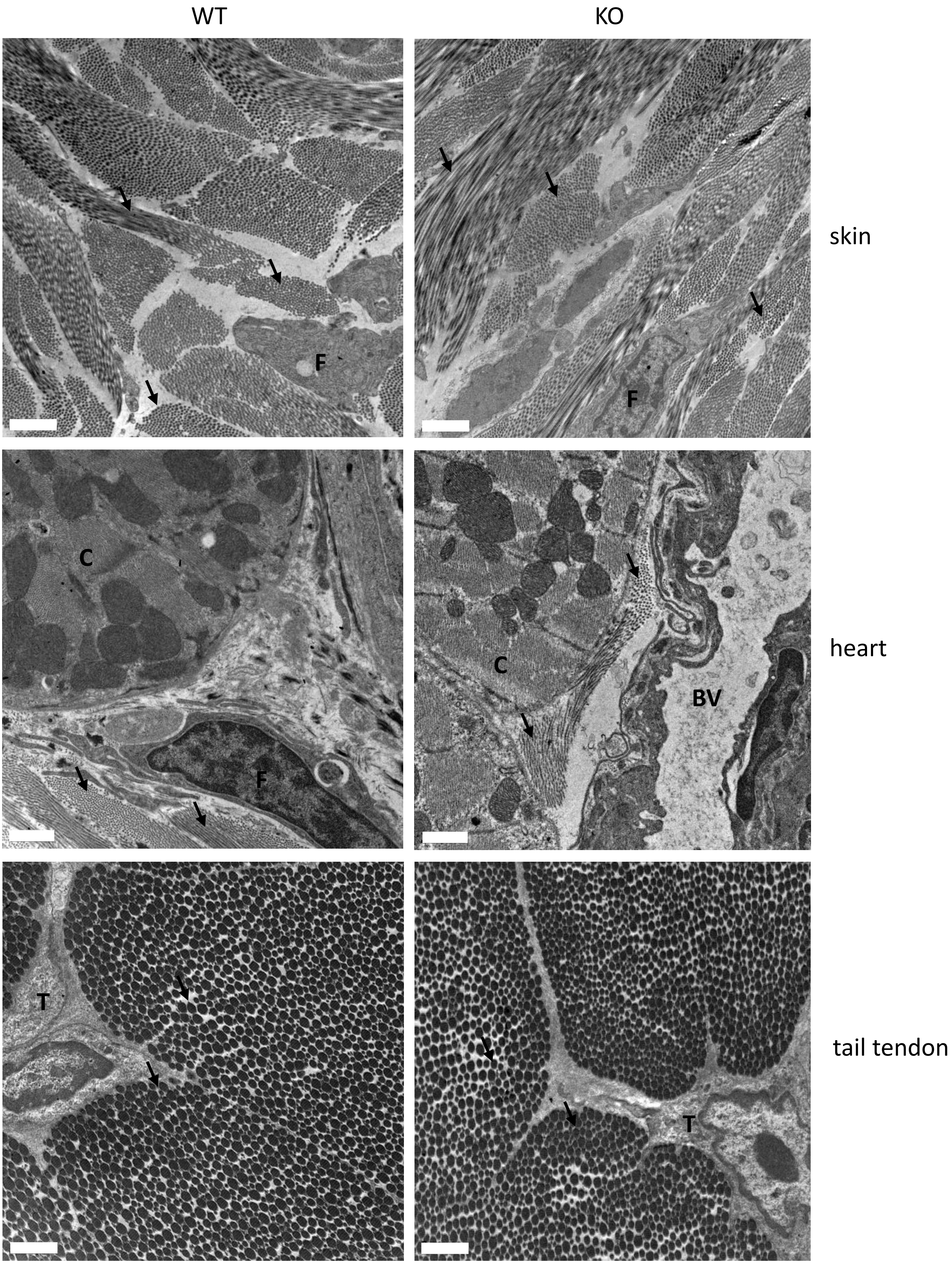

Supplementary Figure 2: Transmission electron microscopy pictures of collagen fibrils in WT and *Pcolce2*-null (KO) mice. Conditions are the same as in Fig. 1a for skin but lower magnifications are shown. For heart and tail tendons, images were also obtained from 6 week-old animals. Bars correspond to 1 µm for heart and 2 µm for skin and tail tendon. Black arrows indicate collagen fibrils in longitudinal or transverse sections. F, fibroblast; C, cardiomyocyte; BV, lumen of a blood vessel; T, tenocyte.

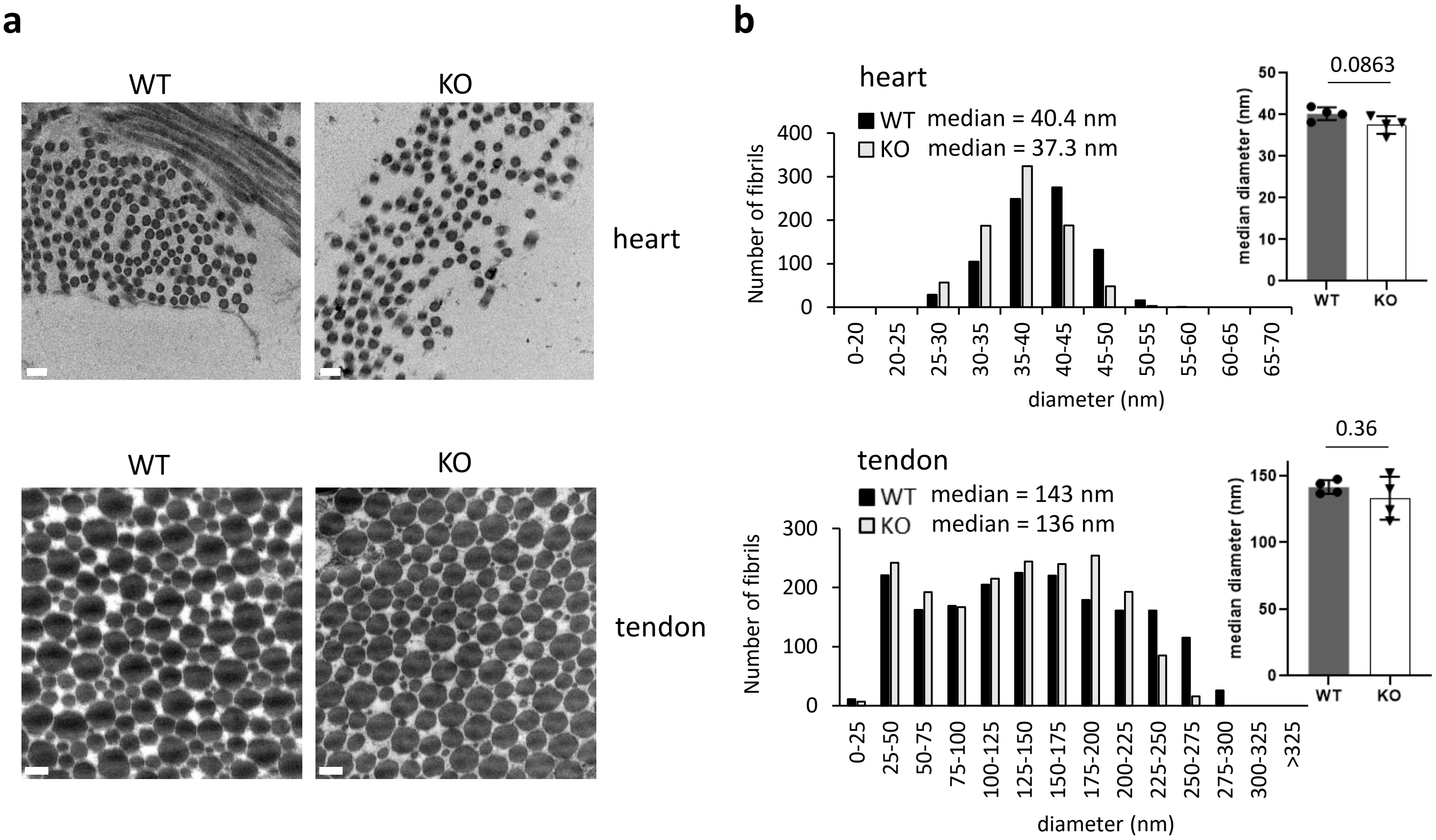

Supplementary Figure 3: PCPE-2 has no major impact on the organization of collagen fibrils in the heart and tail tendon. a TEM pictures of the collagen fibrils in heart and tail tendon of 6 week-old WT and KO mice. Representative images of n = 4 mice for each genotype. Bar = 100 nm (heart) or 200 nm (tail tendon). **b** Distributions of collagen fibril diameters for WT and KO mice and median values of all measurements; 807 fibrils from n = 4 mice were analyzed for each genotype in heart and 1855 fibrils from n = 4 mice in tail tendon. The graph of the median diameters calculated for individual mice is also shown (means ± SD; n = 4 mice/genotype; unpaired two-sided t-test).

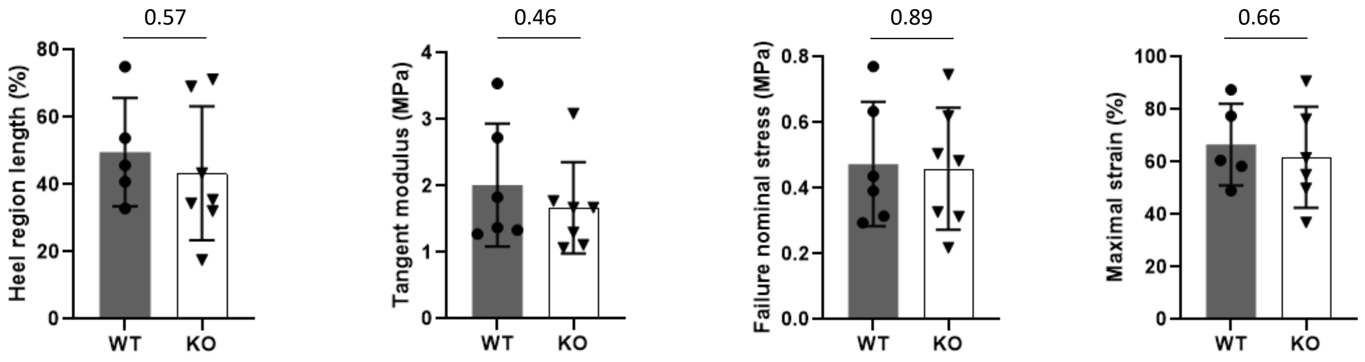

Supplementary Figure 4: Comparison of stretching parameters for the skin of WT and KO mice. Data show means ± SD of n = 6 (or 5 for heel region length and maximal strain measurements) mice for WT and n = 7 mice for KO (age of the mice: 8 weeks; unpaired two-sided t-test).

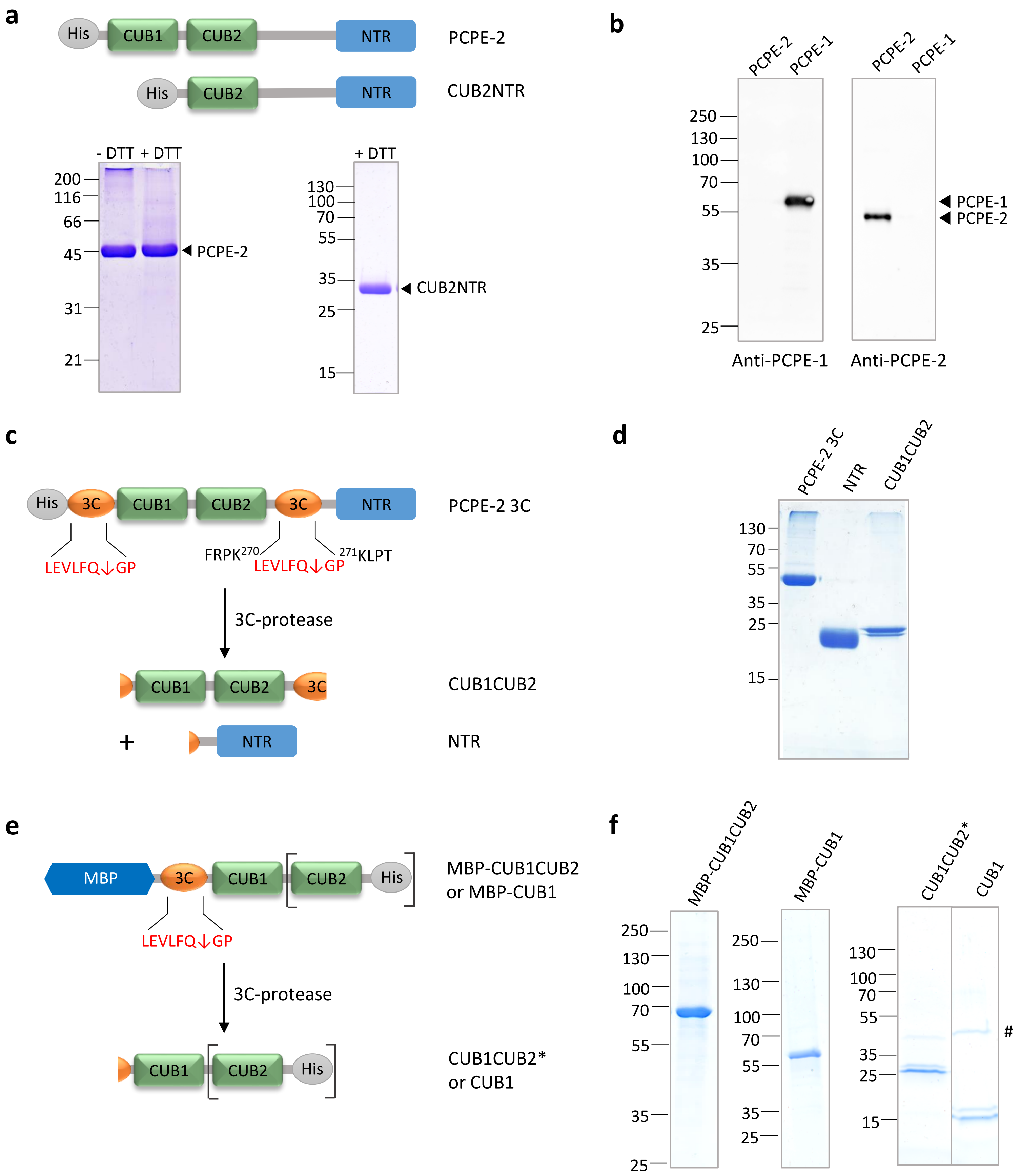

Supplementary Figure 5: Purity of recombinant human PCPE-2 and strategies to obtain the CUB2NTR, CUB1CUB2, NTR and CUB1 domains of PCPE-2. a Domain structures of recombinant PCPE-2 and CUB2NTR constructs and SDS-PAGE analysis of purified proteins in reducing (+ DTT) or non-reducing (- DTT) conditions. **b** Immunodetection of PCPE-1 and PCPE-2 in 250 ng of each recombinant protein using the AF2627 antibody to detect PCPE-1 and the home-made antibody to detect PCPE-2 (reducing conditions). **c** Domain structure of the PCPE-2 3C construct and strategy to obtain the CUB1CUB2 and NTR domains of PCPE-2. The cleavage sequence of the HRV 3C-protease and the position of the insertion site in the linker between the CUB1CUB2 and NTR domains of human PCPE-2 are indicated. **d** SDS-PAGE analysis of the resulting purified proteins in non-reducing conditions. The two bands observed for CUB1CUB2 are thought to correspond to distinct O-glycosylated forms. **e** Domain structure of the MBP-CUB1CUB2 and MBP-CUB1 constructs and strategy to obtain CUB1CUB2 (* indicates the presence of a 6His tag) or the isolated CUB1 domain from these fusion proteins. **f** SDS-PAGE analysis of the resulting purified proteins in reducing conditions (# indicates the presence of residual cleaved MBP).

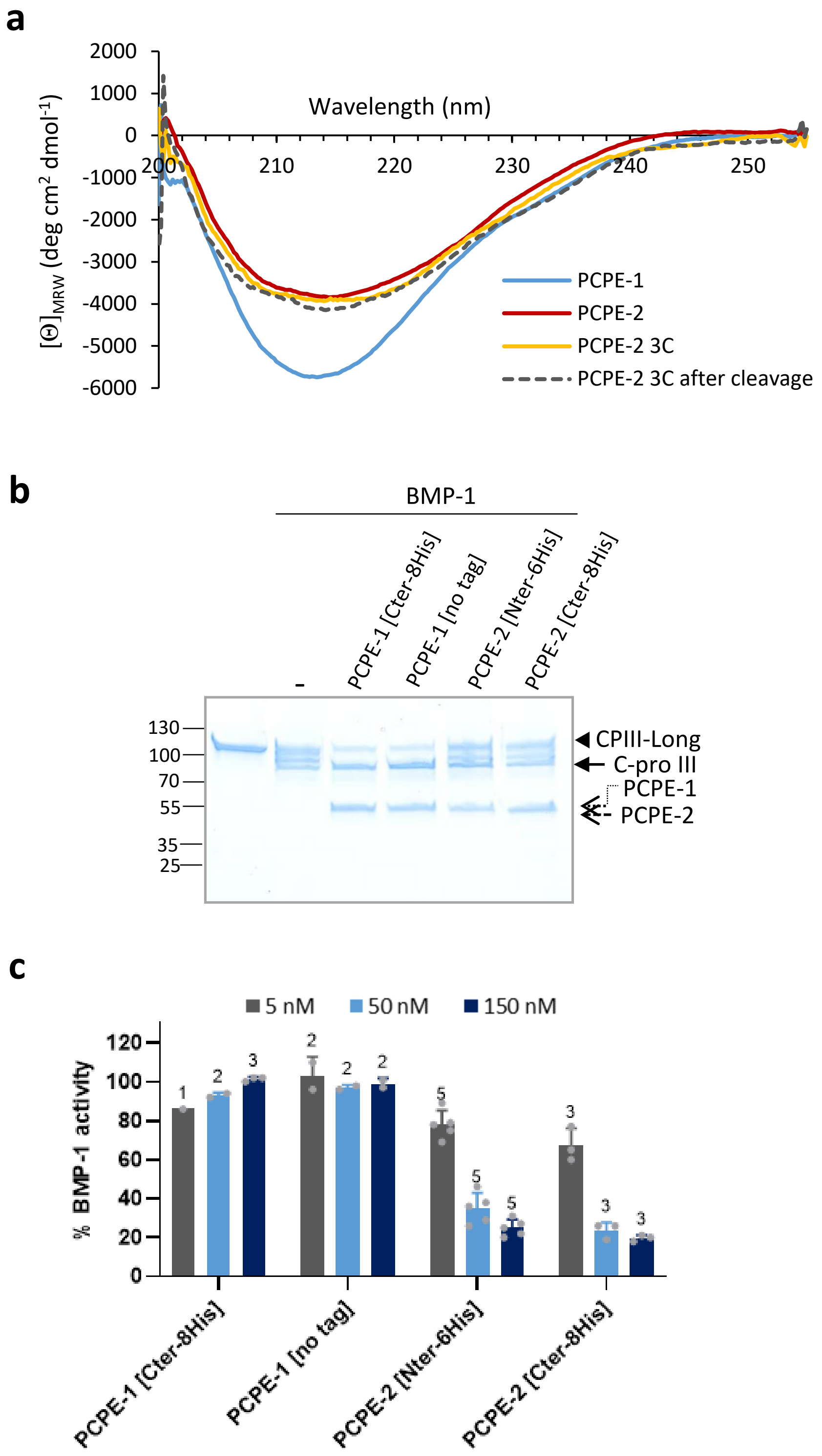

Supplementary Figure 6: Quality control of PCPE constructs. a CD spectra of 200 µg/ml PCPE-1, PCPE-2, PCPE-2 3C and PCPE-2 3C after overnight cleavage by HRV 3C protease. **b** Effect of PCPE-1 (with no tag or a 8His tag at the C-terminus) or PCPE-2 (with 6His tag at the N-terminus, as used this study, or with a 8His tag at the C-terminus) on the cleavage of CPIII-Long by BMP-1 in the same assay conditions as in Fig. 2b. The gel is representative of n = 3 independent experiments. **c** Quantification of BMP-1 activity on the fluorogenic peptide Mca-YVADAPK(Dnp)-OH in the absence or presence of increasing concentrations of the same PCPE constructs as in b. Same conditions as in Figure 4a. Means ± SD (number of independent experiments run in duplicate indicated above each bar).

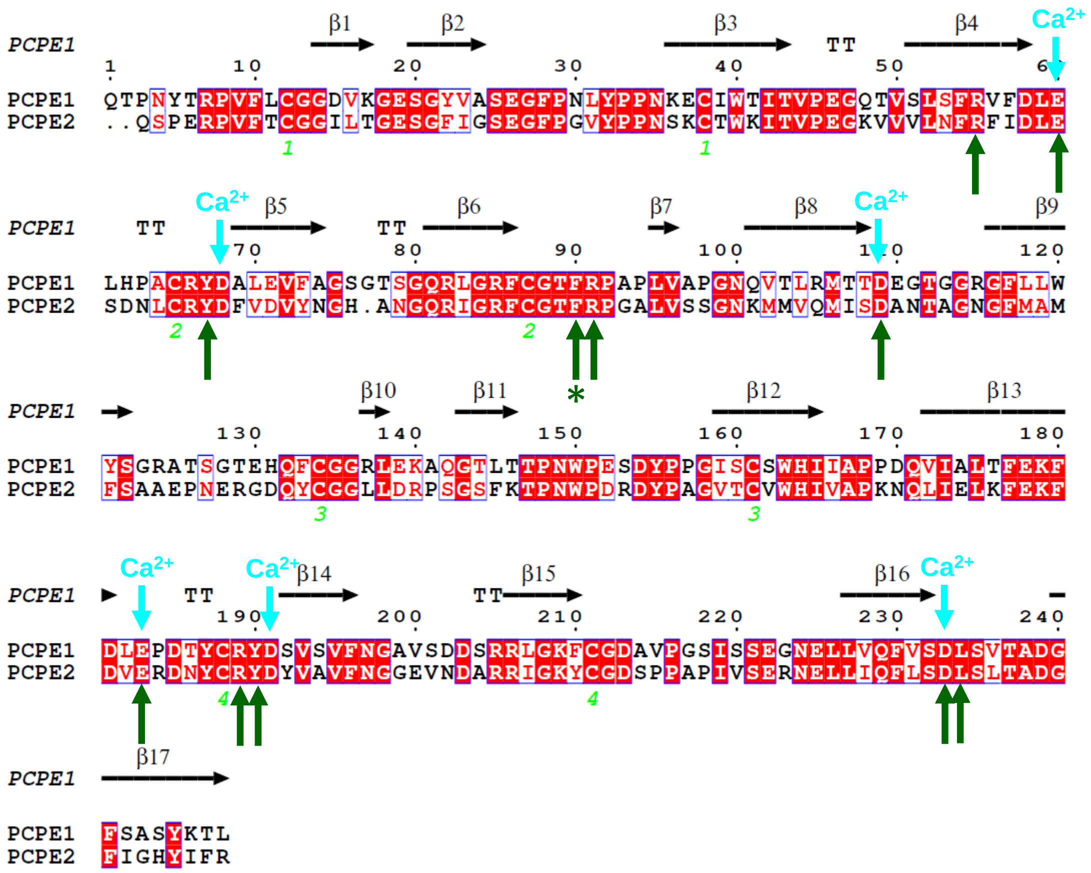

Supplementary Figure 7: Alignment of the CUB domains of human PCPE-1 and PCPE-2 showing the conservation of the residues involved in the interaction with the C-propeptide of procollagen III. Numbering refers to mature human PCPE-1. CUB1: residues 12-123, CUB2: residues 132-248. Identical residues are indicated by white labels on a red background, conservative changes by red on white and non-conservative changes by black on white (identity between the two sequences = 54.3 %). Secondary structures from PDB entry 6FZV. Key residues involved in the interaction of PCPE-1 with procollagen III C-propeptide are indicated with green arrows. Calcium ligands (blue arrows), cysteines involved in disulphide bridges (green numbers) and the conserved phenylalanine mutated in the experiments shown in Fig. 5 (F87 in PCPE-2; *) are also indicated.

a

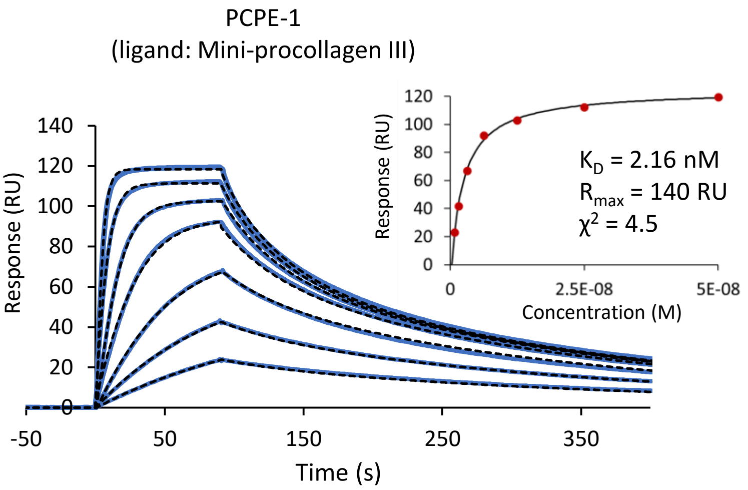

Heterogeneous ligand model	k_{a1} ($M^{-1}s^{-1}$)	k_{d1} (s^{-1})	**K_{D1} (nM)**	R_{max1} (%)	χ^2
	$7.9\ 10^6$	0.0043	**0.55**	61	
**PCPE-1**	k_{a2} ($M^{-1}s^{-1}$)	k_{d2} (s^{-1})	**K_{D2} (nM)**	R_{max2} (%)	0.43
	$4.8\ 10^6$	0.026	**5.4**	39	

b

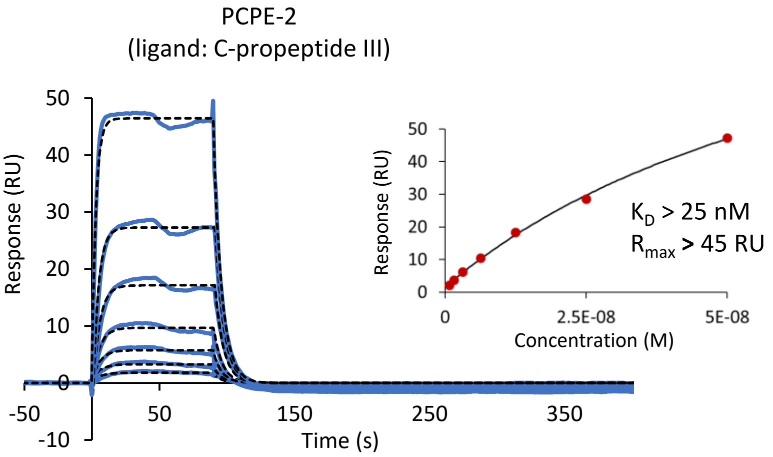

1:1 binding model	k_{a1} ($M^{-1}s^{-1}$)	k_{d1} (s^{-1})	**K_{D1} (nM)**	R_{max1} (RU)	χ^2
**PCPE-2**	$3.6\ 10^6$	0.16	**45**	82	0.91

c

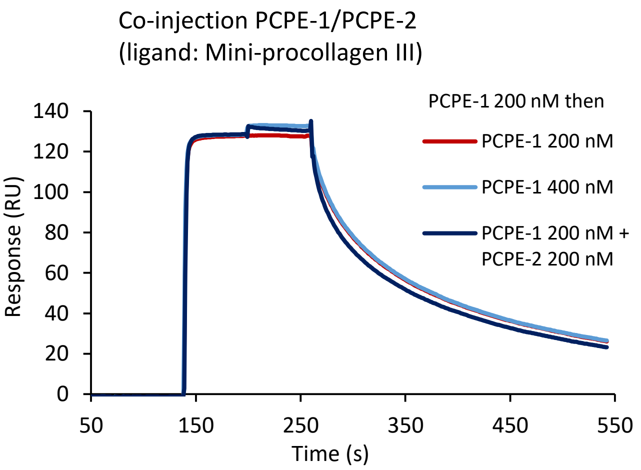

Supplementary Figure 8: The interaction between PCPE-2 and mini-procollagens relies on the same domains as the interaction between PCPE-1 and mini-procollagens. a PCPE-1 binding to immobilized mini-procollagen III (435 RU). Increasing concentrations of PCPE-1 were injected (0.78-50 nM, prepared as serial two-fold dilutions) and fits were obtained with the kinetic (black dotted lines; heterogeneous ligand) and steady-state (inset) models. The main parameters derived from the best fits obtained in the two conditions are also indicated. **b** Fits of the sensorgrams obtained in the same conditions when PCPE-2 was injected over immobilized C-propeptide III (351 RU). Best fit of the kinetic data was obtained here with the 1:1 binding model. **c** Successive injections of PCPE-1 + PCPE-2 on immobilized mini-procollagen III (435 RU) compared to the successive injections of PCPE-1 alone.

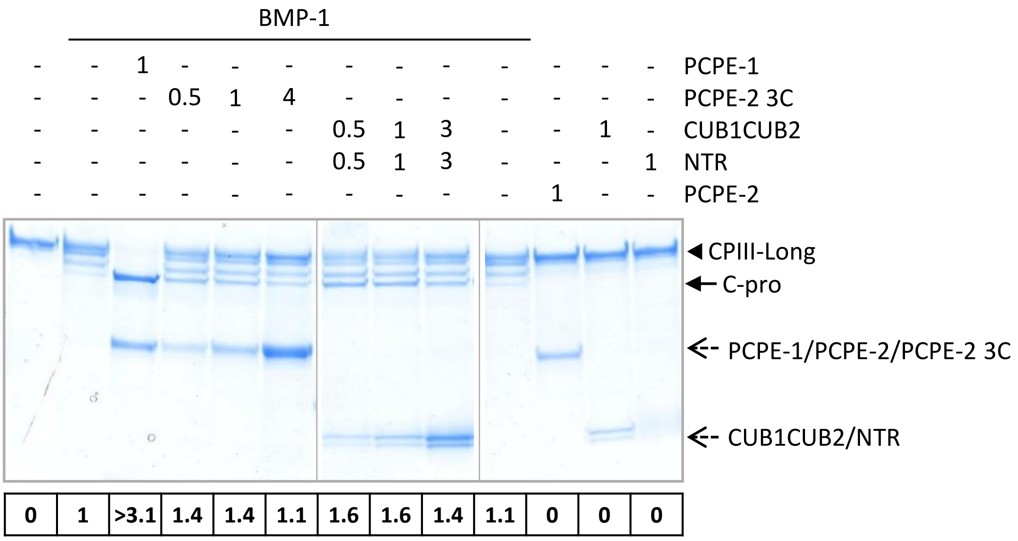

Supplementary Figure 9: PCPE-2 3C and the mixture of CUB1CUB2 and NTR domains have the same effects on BMP-1 PCP activity as PCPE-2 and CUB1CUB2 alone respectively. Cleavage of CPIII-Long (360 nM) by BMP-1 (20 nM) in the absence or presence of PCPE-1, PCPE-2 3C or a mixture of the CUB and NTR domains of PCPE-2 for 1 h. In the absence of BMP-1, CPIII-Long is not affected by PCPE-2 nor its domains (last three lanes). Molar ratios of PCPEs and domains to CPIII-Long are indicated above the gel and the enhancement factors are indicated below the gel. The gel is representative of n = 3 independent experiments and an uncropped version is available in Supplementary Fig. 16 and as a Source Data file.

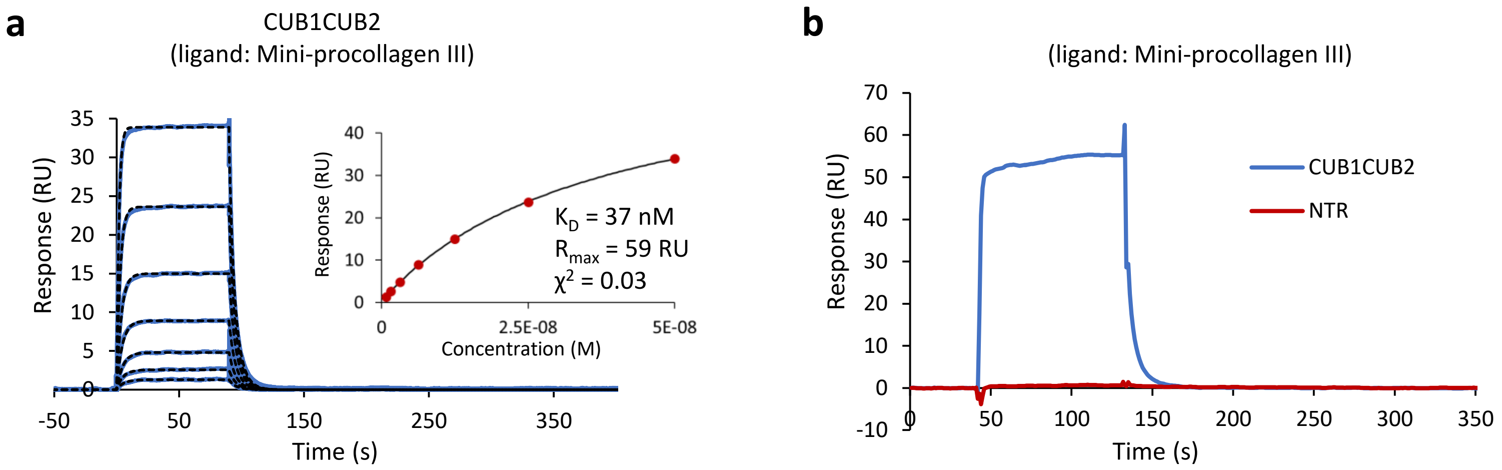

1:1 binding model	k_{a1} ($M^{-1}s^{-1}$)	k_{d1} (s^{-1})	**K_{D1} (nM)**	R_{max1} (RU)	χ^2
**CUB1CUB2 (PCPE-2)**	9.7 10^6	0.30	**31**	52	0.02

Supplementary Figure 10: Interactions of the CUB1CUB2 and NTR domains of PCPE-2 with procollagens. a Fits of the sensorgrams obtained with kinetic (black dotted lines; 1:1 binding) or steady-state (inset) models when increasing concentrations of the CUB1CUB2 domains of PCPE-2 were injected (0.78-50 nM, prepared as serial two-fold dilutions) over immobilized mini-procollagen III (435 RU). **b** Comparison of the binding of 200 nM of the CUB1CUB2 and NTR domains of PCPE-2 on immobilized mini-procollagen III (435 RU).

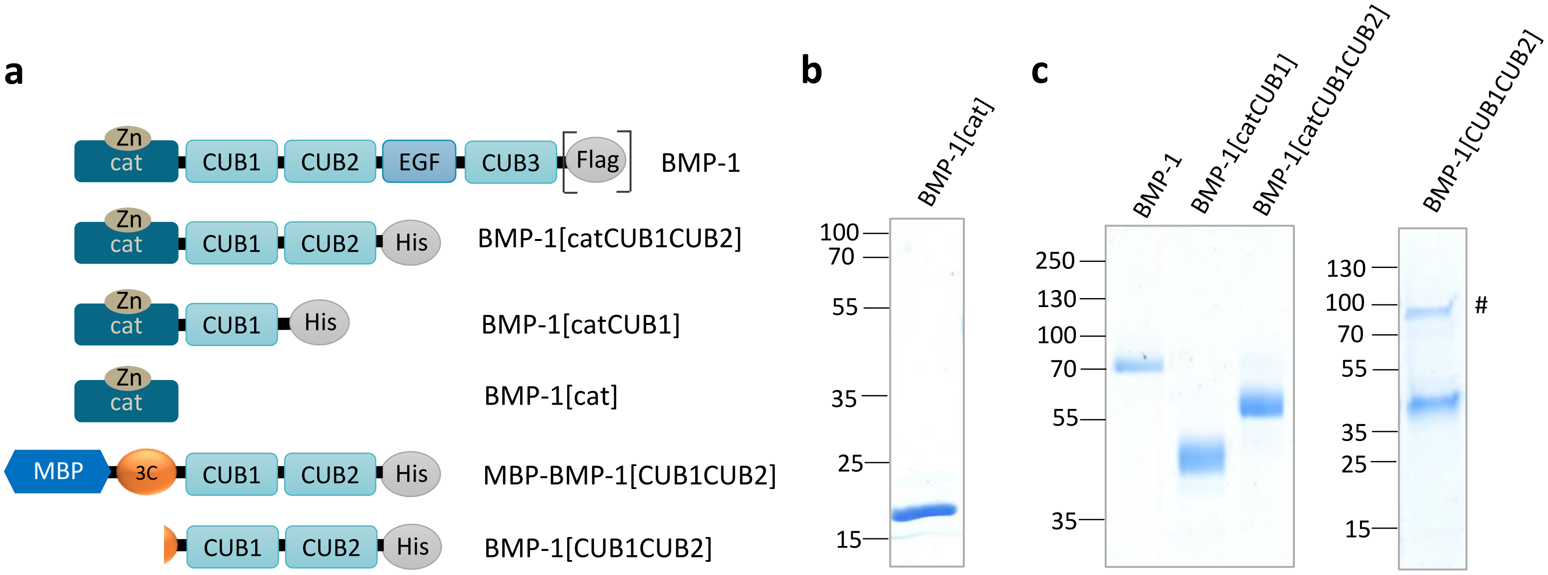

Supplementary Figure 11: Structure and purity of recombinant human BMP-1 and deletion mutants. a Domain composition of BMP-1-derived proteins. Note that the Flag tag of BMP-1 is mostly lost during production and purification. **b** SDS-PAGE analysis of the purified catalytic domain of human BMP-1 produced in *E. Coli*. **b** SDS-PAGE analysis of BMP-1 and its deletion mutants produced in HEK 293 cells. Note that incomplete cleavage of MBP-BMP-1[CUB1CUB2] by HRV 3C-protease leads to the presence of some residual fusion protein in the preparation of BMP-1[CUB1CUB2] (around 25 %, indicated by #).

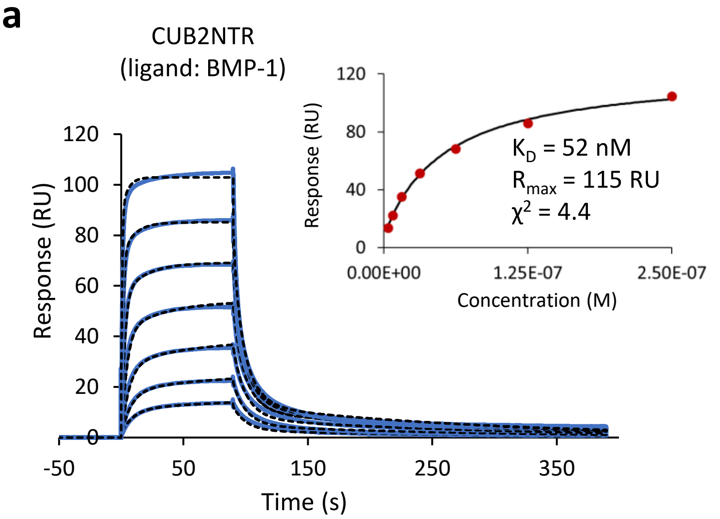

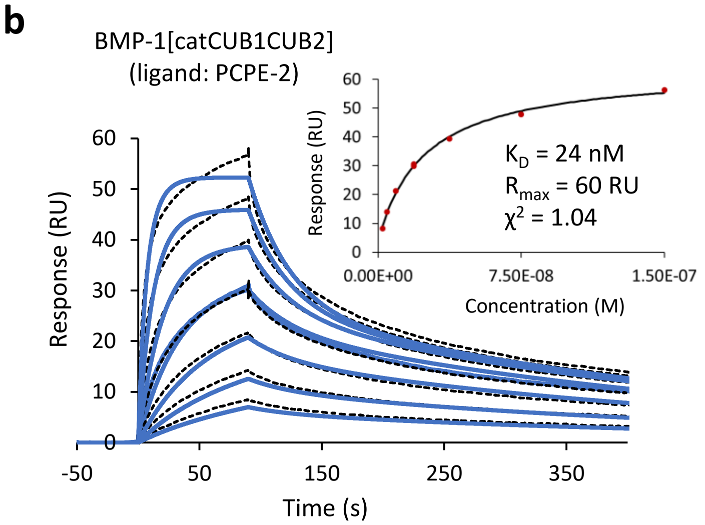

Heterogeneous ligand model	k_{a1} ($M^{-1}s^{-1}$)	k_{d1} (s^{-1})	**K_{D1} (nM)**	R_{max1} (%)	χ^2
**CUB2NTR (PCPE-2)**	$7.9\ 10^5$	0.005	**6.29**	14	0.56
	k_{a2} ($M^{-1}s^{-1}$)	k_{d2} (s^{-1})	**K_{D2} (nM)**	R_{max2} (%)	
	$7.4\ 10^6$	0.198	**26.6**	86	

Heterogeneous ligand model	k_{a1} ($M^{-1}s^{-1}$)	k_{d1} (s^{-1})	**K_{D1} (nM)**	R_{max1} (%)	χ^2
**BMP-1 [catCUB1CUB2]**	$1.3\ 10^6$	0.0024	**1.84**	42	0.96
	k_{a2} ($M^{-1}s^{-1}$)	k_{d2} (s^{-1})	**K_{D2} (nM)**	R_{max2} (%)	
	$4.5\ 10^5$	0.030	**66**	58	

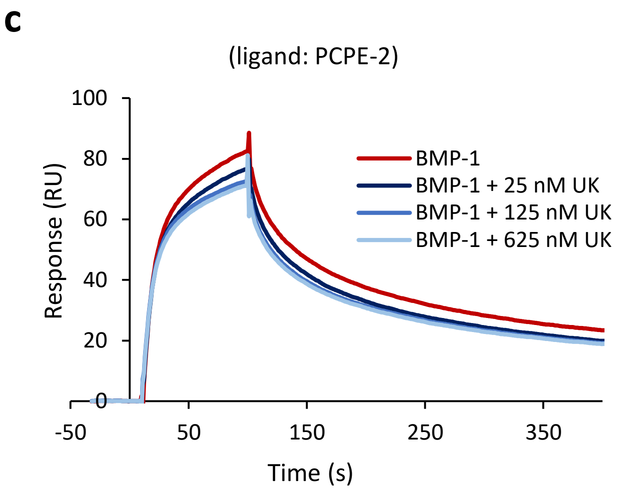

Supplementary Figure 12: Identification of the domains involved in the interaction between PCPE-2 and BMP-1. a Fits of the sensorgrams obtained with kinetic (black dotted lines; heterogenous ligand) or steady-state (inset) models when increasing concentrations of the CUB2NTR domains of PCPE-2 (3.9-250 nM, prepared as serial two-fold dilutions) were injected over immobilized BMP-1 (974 RU). **b** Fits of the sensorgrams obtained with kinetic (black dotted lines; heterogenous ligand) or steady-state (inset) models when increasing concentrations of the BMP-1[catCUB1CUB2] construct (2.3-150 nM, prepared as serial two-fold dilutions) were injected over immobilized PCPE-2 (468 RU). **c** Interaction of 25 nM BMP-1 with PCPE-2 in the presence of increasing concentrations of a synthetic inhibitor binding into BMP-1 active site (UK 383,367).

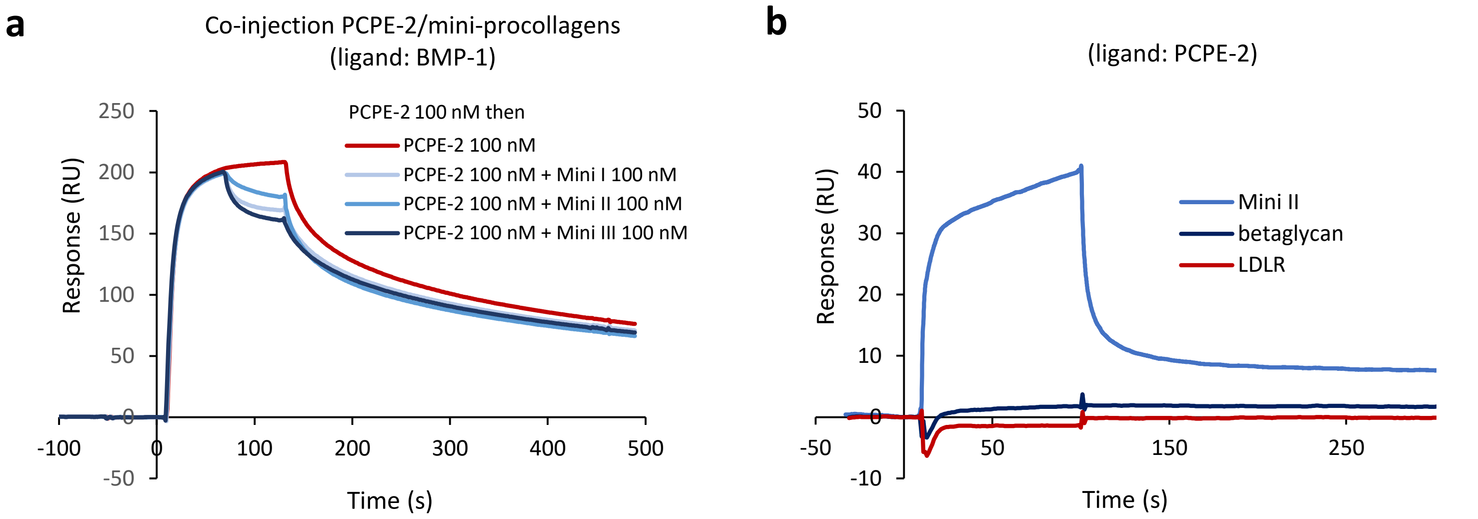

Supplementary Figure 13: Interactions of PCPE-2 with BMP-1 substrates. a Successive injections of PCPE-2 + mini-procollagen I, II or III on immobilized BMP-1 (435 RU) compared to the successive injections of PCPE-2 alone. **b** Comparison of the binding of 75 nM mini-procollagen II (Mini II, MW = 105 kDa) with the binding of 100 nM betaglycan (MW = 81 kDa) and 100 nM of the ectodomain of LDLR (MW = 86 kDa) on immobilized PCPE-2 (468 RU).

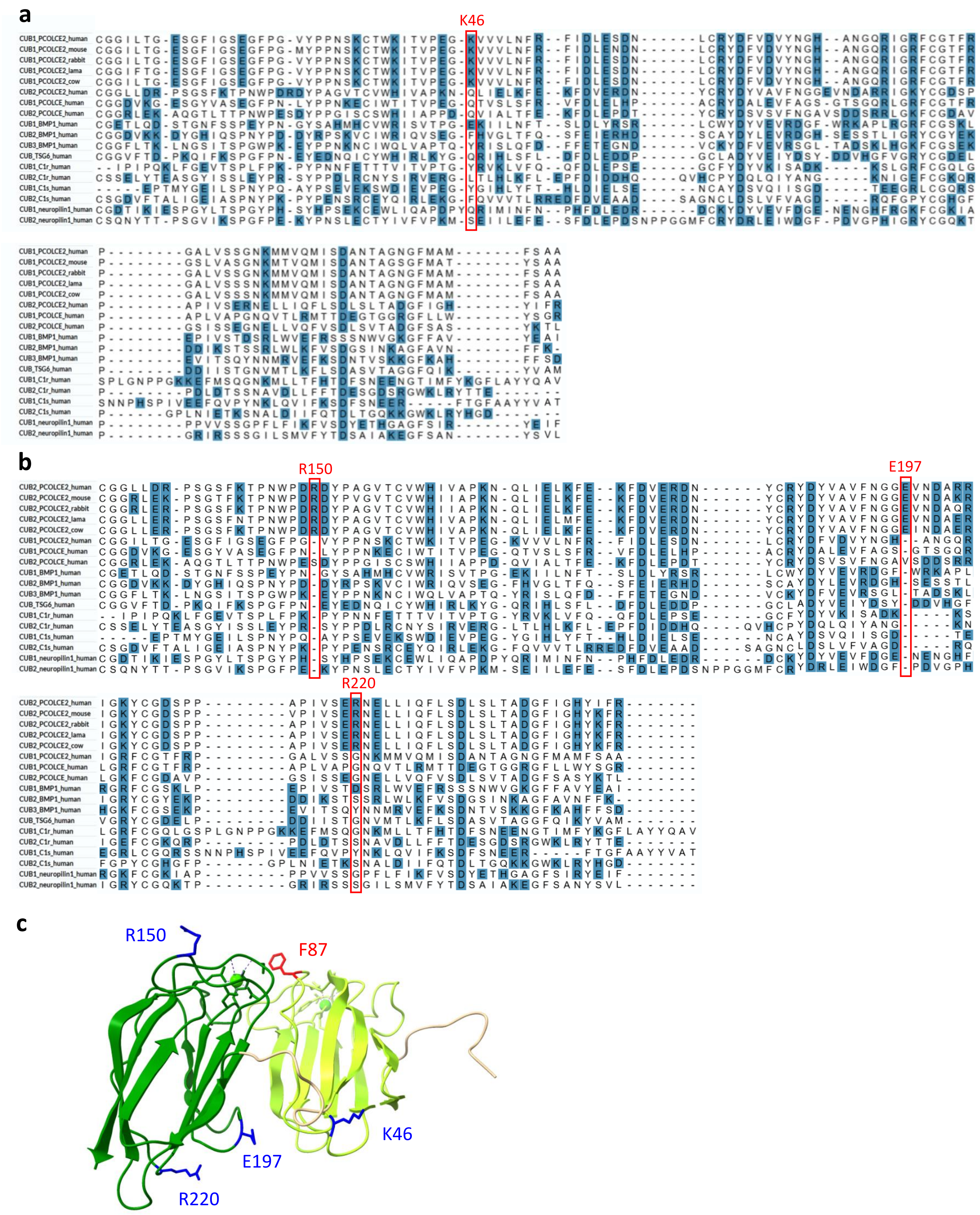

Supplementary Figure 14: Alignment of the CUB domains of human PCPE-2 with a selection of other CUB domains highlighting charged residues. a The CUB1 domain of PCPE-2 from 5 different species was compared to other CUB domains. **b** The CUB2 domain of PCPE-2 from 5 different species was compared to other CUB domains. Conserved and charged residues that are unique to the CUB domains of PCPE-2 and not located in β-sheets are marked in red. Alignements were obtained with Clustal Omega. **c** Positions of mutated amino-acids on the model of the CUB domains of PCPE-2 obtained as described in Fig. 2d (CUB1 in light green, CUB2 in dark green).

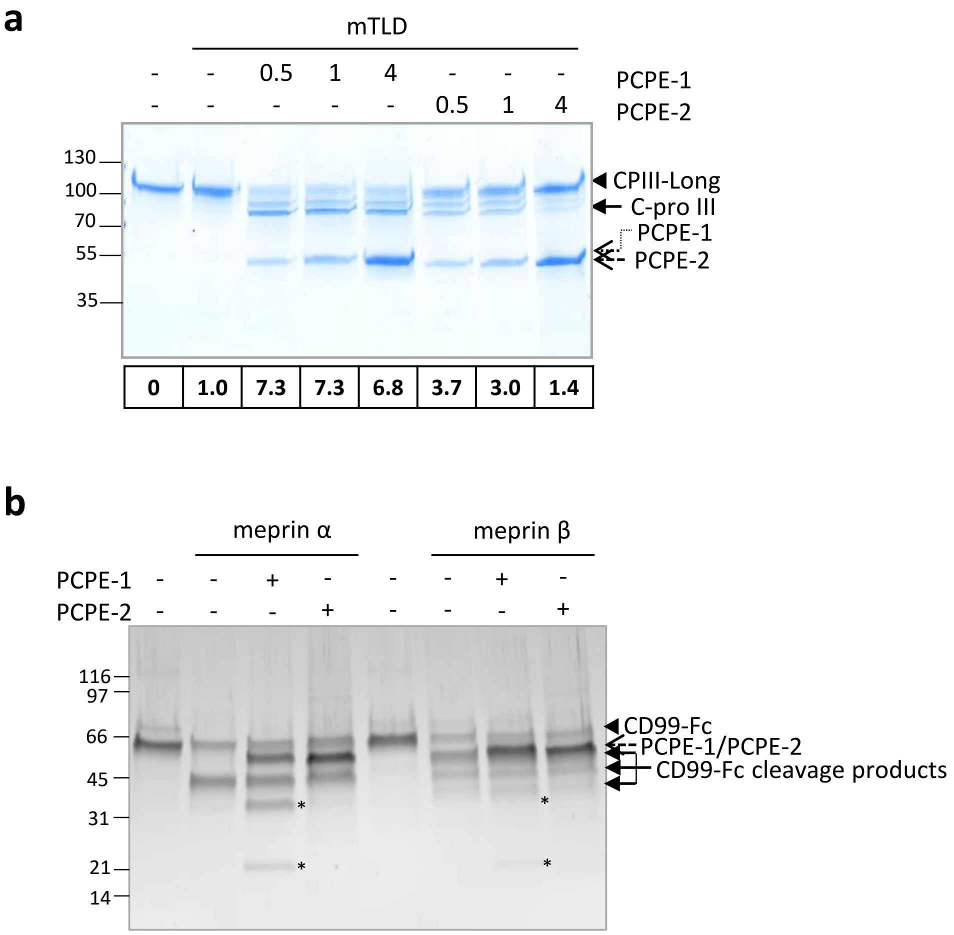

Supplementary Figure 15: Effect of PCPE-2 on the cleavage of CPIII-Long by mTLD and of CD99 by meprins. a SDS-PAGE analysis of the cleavage of CPIII-Long (330 nM) by mTLD (22 nM) in the absence or presence of PCPE-1 or PCPE-2 for 2 h (non-reducing conditions). Molar ratios of PCPEs to CPIII-Long are indicated above the gel and the enhancement factors are indicated below the gel. The gel is representative of n = 3 independent experiments. **b** SDS-PAGE analysis in non-reducing conditions of the cleavage of the recombinant ectodomain of human CD99 (fused to the Fc region of human IgG; 500 nM) by meprin α (1 nM) or meprin β (0.33 nM) for 30 min. When present, PCPE-1 and PCPE-2 were added at the same concentration as the substrate (500 nM; molar ratio to protease ≥ 500). Detection was with Sypro Ruby staining and (*) indicates PCPE-1 cleavage products generated by meprins. The gel is representative of n = 2 independent experiments.

Fig. 1d (LF41 antibody)

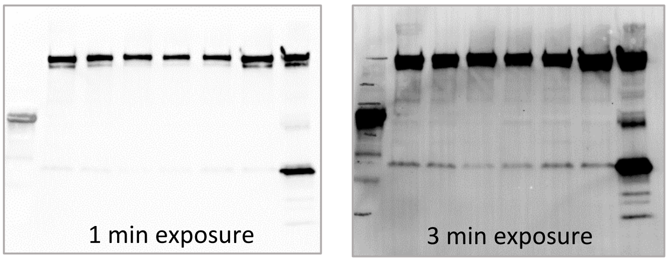

Fig. 1e (LF41 antibody)

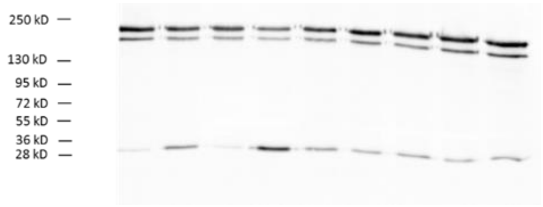

Fig. 3b (Coomassie Blue)

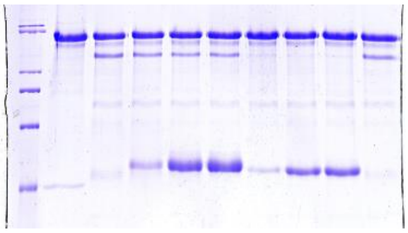

Fig. 3c (Coomassie Blue)

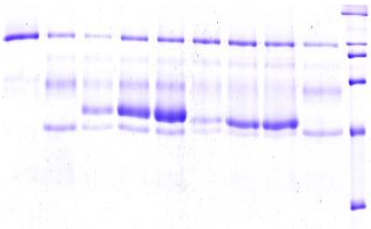

Fig. 3d (Coomassie Blue)

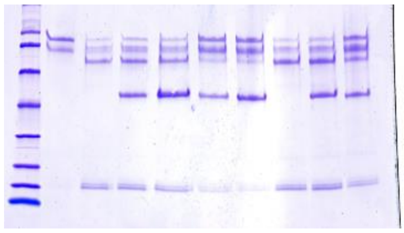

Fig. 3f (Sypro Ruby)

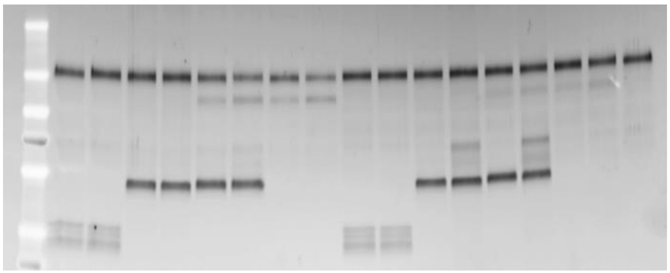

Supplementary Fig. 5f (Sypro Ruby)

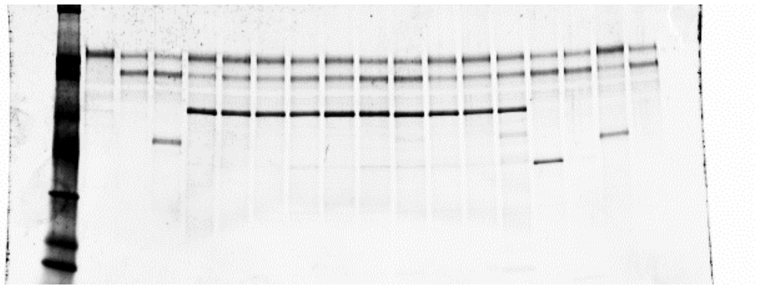

Fig. 6c (Instant Blue)

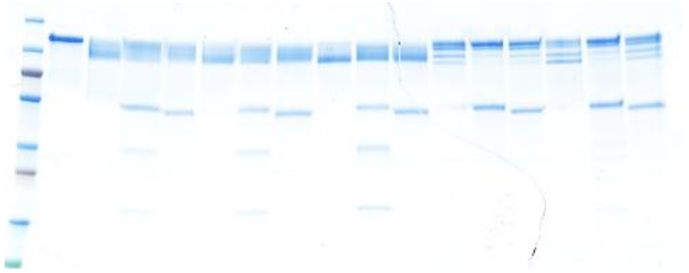

Supplementary Fig. 9 (Instant Blue)

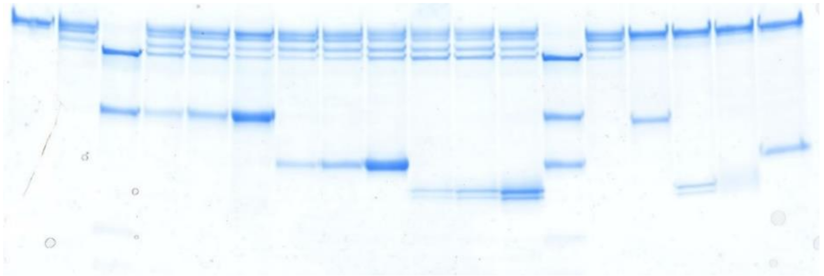

Supplementary Figure 16: Uncropped gels and immunoblots. Refer to the indicated figures for the corresponding legends.

Supplementary references

1. Hung, C. W. *et al.* Characterization of post-translational modifications in full-length human BMP-1 confirms the presence of a rare vicinal disulfide linkage in the catalytic domain and highlights novel features of the EGF domain. *J Proteomics* **138**, 136-145 (2016).
2. Blanc, G. *et al.* Insights into how CUB domains can exert specific functions while sharing a common fold: Conserved and specific features of the CUB1 domain contribute to the molecular basis of procollagen C-proteinase enhancer-1 activity. *J Biol Chem* **282**, 16924-16933 (2007).
3. Moali, C. *et al.* Substrate-specific modulation of a multisubstrate proteinase. C-terminal processing of fibrillar procollagens is the only BMP-1-dependent activity to be enhanced by PCPE-1. *J Biol Chem* **280**, 24188-24194 (2005).
4. Bekhouche, M. *et al.* Role of the netrin-like domain of procollagen C-proteinase enhancer-1 in the control of metalloproteinase activity. *J Biol Chem* **285**, 15950-15959 (2010).
5. Vadon-Le Goff, S. *et al.* Procollagen C-proteinase enhancer stimulates procollagen processing by binding to the C-propeptide region only. *J Biol Chem* **286**, 38932-38938 (2011).
6. Bonod-Bidaud, C. *et al.* Enzymatic cleavage specificity of the proalpha1(V) chain processing analysed by site-directed mutagenesis. *Biochem J* **405**, 299-306 (2007).
7. Bourhis, J. M. *et al.* Procollagen C-proteinase enhancer grasps the stalk of the C-propeptide trimer to boost collagen precursor maturation. *Proc Natl Acad Sci USA* **110**, 6394-6399 (2013).
8. Mariano, N. *et al.* Production of recombinant heterotrimeric mini-procollagen I and homotrimeric mini-procollagen II reveals new cleavage sites for BMP-1. *BioRxiv* **10.1101/2022.11.10.516045** (2022).
9. Pulido, D. *et al.* Structural Basis for the Acceleration of Procollagen Processing by Procollagen C-Proteinase Enhancer-1. *Structure* **26**, 1384-1392 e1383 (2018).
10. Vilchis-Landeros, M. M., Montiel, J. L., Mendoza, V., Mendoza-Hernandez, G. & Lopez-Casillas, F. Recombinant soluble betaglycan is a potent and isoform-selective transforming growth factor-beta neutralizing agent. *Biochem J* **355**, 215-222 (2001).
11. Becker, C. *et al.* Differences in the activation mechanism between the alpha and beta subunits of human meprin. *Biol Chem* **384**, 825-831 (2003).
12. Becker-Pauly, C. *et al.* The alpha and beta subunits of the metalloprotease meprin are expressed in separate layers of human epidermis, revealing different functions in keratinocyte proliferation and differentiation. *J Invest Dermatol* **127**, 1115-1125 (2007).
13. Anastasi, C. *et al.* BMP-1 disrupts cell adhesion and enhances TGF-beta activation through cleavage of the matricellular protein thrombospondin-1. *Sci Signal* **13**, eaba3880 (2020).
14. Mac Sweeney, A. *et al.* Structural basis for the substrate specificity of bone morphogenetic protein 1/tolloid-like metalloproteases. *J Mol Biol* **384**, 228-239 (2008).
15. Berry, R. *et al.* Role of dimerization and substrate exclusion in the regulation of bone morphogenetic protein-1 and mammalian tolloid. *Proc Natl Acad Sci USA* **106**, 8561-8566 (2009).
