# Supplementary material for: Identification of PCPE-2 as the endogenous specific inhibitor of human BMP-1/tolloid-like proteinases
Source: Nat Commun. 2023 Dec 4;14:8020. doi: 10.1038/s41467-023-43401-0 (PMC10696041; doi:10.1038/s41467-023-43401-0)
Supplement: Supplementary file 3 — Reporting Summary [file 41467_2023_43401_MOESM3_ESM.pdf]

nature portfolio

Corresponding author(s): Catherine Moali

Last updated by author(s): Oct 18, 2023

Reporting Summary

Nature Portfolio wishes to improve the reproducibility of the work that we publish. This form provides structure for consistency and transparency in reporting. For further information on Nature Portfolio policies, see our Editorial Policies and the Editorial Policy Checklist.

Statistics

For all statistical analyses, confirm that the following items are present in the figure legend, table legend, main text, or Methods section.

n/a	Confirmed	
☐	☒	The exact sample size (*n*) for each experimental group/condition, given as a discrete number and unit of measurement
☐	☒	A statement on whether measurements were taken from distinct samples or whether the same sample was measured repeatedly
☐	☒	The statistical test(s) used AND whether they are one- or two-sided<br>*Only common tests should be described solely by name; describe more complex techniques in the Methods section.*
☒	☐	A description of all covariates tested
☐	☒	A description of any assumptions or corrections, such as tests of normality and adjustment for multiple comparisons
☐	☒	A full description of the statistical parameters including central tendency (e.g. means) or other basic estimates (e.g. regression coefficient) AND variation (e.g. standard deviation) or associated estimates of uncertainty (e.g. confidence intervals)
☐	☒	For null hypothesis testing, the test statistic (e.g. *F*, *t*, *r*) with confidence intervals, effect sizes, degrees of freedom and *P* value noted<br>*Give P values as exact values whenever suitable.*
☒	☐	For Bayesian analysis, information on the choice of priors and Markov chain Monte Carlo settings
☒	☐	For hierarchical and complex designs, identification of the appropriate level for tests and full reporting of outcomes
☒	☐	Estimates of effect sizes (e.g. Cohen's *d*, Pearson's *r*), indicating how they were calculated

Our web collection on statistics for biologists contains articles on many of the points above.

Software and code

Policy information about availability of computer code

Data collection

Digital Micrograph software v1.7 (Gatan Inc)
Biacore T200 control software v3.2.1 (Cytiva)
FusionCapt Advance FX 16.16b (Vilber Lourmat)
CFX Manager Software (Biorad)
Rotor-Gene Q software v2.3.5 (Qiagen)
Typhoon FLA 9500 control software (Cytiva)
i-control software v1.10 (Tecan)
Yasara (http://www.yasara.org)
Chirascan software (Applied Photophysics)

Data analysis

Excel 2016 or 2019 (Microsoft)
GraphPad Prism 8 or 9
CorrelManuV v1.681
ImageQuant TL software v8.2 (Cytiva)
FusionCapt Advance FX 7 16.16b (Vilbert Lourmat)
Biacore T200 evaluation software v3.2.1 (Cytiva)
ImageJ v1.53
Clustal Omega (https://www.uniprot.org/align/)
ESPript 3.0 (https://espript.ibcp.fr)
UCSF Chimera (https://www.cgl.ucsf.edu/chimera) or ChimeraX (http://www.rbvi.ucsf.edu/chimerax)

For manuscripts utilizing custom algorithms or software that are central to the research but not yet described in published literature, software must be made available to editors and reviewers. We strongly encourage code deposition in a community repository (e.g. GitHub). See the Nature Portfolio guidelines for submitting code & software for further information.

Data

Policy information about availability of data

All manuscripts must include a data availability statement. This statement should provide the following information, where applicable:
- Accession codes, unique identifiers, or web links for publicly available datasets
- A description of any restrictions on data availability
- For clinical datasets or third party data, please ensure that the statement adheres to our policy

The structural data used in this study are available in the Protein Data Bank under accession code 6FZV [https://doi.org/10.2210/pdb6FZV/pdb].

Human research participants

Policy information about studies involving human research participants and Sex and Gender in Research.

Reporting on sex and gender: Human fibroblasts were extracted from female donors. Not enough male donors could be recruited to be included in this study. Sex was determined by self-reporting.

Population characteristics: Healthy women aged 25-35 years undergoing breast or abdominal plastic surgery were recruited between 2005 and 2014 by investigators of the Cell and Tissue Bank of Edouard Herriot Hospital (Lyon, France) or the Department of Dermatology, Medical Center of the University of Freiburg (Freiburg, Germany).

Recruitment: No selection was performed except for age. There was no self-selection bias or other bias as no grouping was performed.

Ethics oversight: The study was performed in agreement with the principles of the Declaration of Helsinki. Cells were harvested in agreement with the French and German ethical regulations (permanent authorization of the French Ministry of Higher Education, Research and Innovation AC-2019-3476 and ethics committee of the University of Freiburg approval no. 318/18). Donors gave written informed consent for use of the materials for research.

Note that full information on the approval of the study protocol must also be provided in the manuscript.

Field-specific reporting

Please select the one below that is the best fit for your research. If you are not sure, read the appropriate sections before making your selection.

☒ Life sciences ☐ Behavioural & social sciences ☐ Ecological, evolutionary & environmental sciences

For a reference copy of the document with all sections, see nature.com/documents/nr-reporting-summary-flat.pdf

Life sciences study design

All studies must disclose on these points even when the disclosure is negative.

Sample size: No sample size calculations were performed. Quantitative measurements on collagen fibrils were performed with at least 800 fibrils analyzed from a minimum of 4 mice/genotype. Sample size for qRT-PCR and stretching experiments was determined from standards in the field and from previous studies in the lab (n = 6-12 mice/genotype or 4-6 fibroblast donors).

Data exclusions: No data were excluded except if the experiment failed (controls could not be validated).

Replication: Most experiments were performed at least two times. Notable exceptions were the determination of kinetic and steady-state parameters by SPR since the data are based on a high number of curves recorded with a wide range of concentrations (including a duplicate curve) giving highly reliable data. When applicable, all replicate experiments were successful.

Randomization: Randomization is not relevant to this study as no group allocation was performed.

Blinding	Measurements of collagen fibril diameters and quantification of immunoblots was done in blind. Blinding does not apply to quantification based on gels as sample identity is directly visible. Other experiments were quantitative and there was no potential for bias.

Reporting for specific materials, systems and methods

We require information from authors about some types of materials, experimental systems and methods used in many studies. Here, indicate whether each material, system or method listed is relevant to your study. If you are not sure if a list item applies to your research, read the appropriate section before selecting a response.

Materials & experimental systems

n/a	Involved in the study
☐	☒ Antibodies
☐	☒ Eukaryotic cell lines
☒	☐ Palaeontology and archaeology
☐	☒ Animals and other organisms
☒	☐ Clinical data
☒	☐ Dual use research of concern

Methods

n/a	Involved in the study
☒	☐ ChIP-seq
☒	☐ Flow cytometry
☒	☐ MRI-based neuroimaging

Antibodies

Antibodies used	anti-C-propeptide alpha1(I) LF41 antibody : made by Dr. Larry W. Fisher, NIH, Bethesda, USA (Fisher, L. W. et al. Connect Tissue Res 21, 43-48 (1989))<br>anti-human LDLR AF2148 (lot VBC0219101), anti-human PCPE-1 AF2627 (lot XDZ01), anti-mouse PCPE-1 AF2239 (lot YKN01), anti-His tag MAB050 (lot AEJ3320041): all from BioTechne<br>anti-human PCPE-2 : custom-made by Covalab<br>Horse anti-mouse secondary antibody # 7076S (lot 32) and goat anti-rabbit secondary antibody # 7074S (lot 28) : Cell Signaling<br>Donkey anti-goat secondary antibody DkxGt-003-DHRPX (lot 58-72-120417) : ImmunoReagents
Validation	The LF41 is well-described and has been used in the field for more than 30 years.<br>AF2148 : Detects human LDLR in direct ELISAs and Western blots. In direct ELISAs, approximately 15% cross-reactivity with recombinant mouse LDLR is observed (according to manufacturer).<br>AF2627 : Detects human PCPE-1 in direct ELISAs and Western blots. In direct ELISAs, approximately 45% cross-reactivity with recombinant mouse PCPE-1 is observed (according to manufacturer).<br>AF2239 : Detects mouse PCPE-1 in direct ELISAs and Western blots. In direct ELISAs and Western blots, approximately 35% cross-reactivity with recombinant human PCPE is observed (according to manufacturer).<br>MAB050 : Detects proteins containing accessible consecutive histidine regions. The antibody detects His tags localized at the amino- or carboxyl-terminus (according to manufacturer).<br>Anti-human PCPE-2 : Detects human and rat PCPE-2; does not cross-react with human PCPE-1 up to 250 ng (this study).

Eukaryotic cell lines

Policy information about cell lines and Sex and Gender in Research

Cell line source(s)	HEK 293-EBNA cells, HEK 293T cells (modified human embryonic kidney cells, female donor): Cellulonet (SFR Biosciences, Lyon)<br>HEK 293-F cells (modified human embryonic kidney cells, female donor): ThermoFisher Scientific<br>Human skin fibroblasts (female donors, age 25-35 years) : Cell and Tissue Bank of Edouard Herriot Hospital (Lyon, France) or Department of Dermatology, Medical Center of the University of Freiburg (Freiburg, Germany).<br>Mouse skin fibroblasts (newborn female and male animals) : Department of Dermatology, Medical Center of the University of Freiburg (Freiburg, Germany).
Authentication	Primary fibroblasts were authenticated by cell morphology and by western blotting against keratin 14 (negative) and collagen I (positive).
Mycoplasma contamination	HEK 293-EBNA, 293T and 293F cells were tested negative for mycoplasma with the MycoAlert mycoplasma detection kit (Lonza) before use. Skin fibroblasts were screened for mycoplasma contamination by Mycoplasma PCR Detection Test (Thermofisher).
Commonly misidentified lines<br>(See ICLAC register)	No cell lines used are listed in the database of commonly misidentified lines.

Animals and other research organisms

Policy information about studies involving animals; ARRIVE guidelines recommended for reporting animal research, and Sex and Gender in Research

Laboratory animals	Wild-type and Pcolce2-/- mice (Heinzel, K. & Bleul, C. C. The Foxn1-dependent transcripts PCOLCE2 and mPPP1R16B are not required for normal thymopoiesis. Eur J Immunol 37, 2562-2571 (2007)) on C57BL/6 background. Mice between 0-8 weeks of age were used.
Wild animals	The study did not involve wild animals.
Reporting on sex	Equal samples from male and female mice were used.
Field-collected samples	The study did not involve samples collected from the field.
Ethics oversight	All animal procedures were carried out in accordance with the European Directive 2010/63/EU regarding the protection of animals used for scientific purposes. No experimentation on living animals was performed and no specific ethical approval was required.

Note that full information on the approval of the study protocol must also be provided in the manuscript.
